# A Global Pandemic Treaty Must Address Antimicrobial Resistance

**DOI:** 10.1017/jme.2021.94

**Published:** 2021

**Authors:** Lindsay A. Wilson, Susan Rogers Van Katwyk, Isaac Weldon, Steven J. Hoffman

**Keywords:** Antimicrobial Resistance, International Health Regulations, Pandemic Andemic Treaty, World Health Organization, Global Health Law

## Abstract

Antimicrobial resistance (AMR) is one of the defining global health threats of our time, but no international legal instrument currently offers the framework and mechanisms needed to address it. Fortunately, the actions needed to address AMR have considerable overlap with the actions needed to confront other pandemic threats.

The COVID-19 pandemic has demonstrated that no single country can address global health threats alone.^1^ As attention shifts to ensuring better preparedness for future disease outbreaks, a coordinated global strategy will be needed to address future pandemics and mitigate their human, economic, and social toll. International law represents an important tool in this preparedness effort, but existing legal mechanisms lack the coordination and enforcement measures necessary to ensure a coherent and unified pandemic response.^2^ In response to these limitations, members of the World Health Organization agreed in May 2021 to begin discussions about the possibility of a new international pandemic treaty to catalyze collective action against future pandemics.^3^ However, early discussions of the treaty have taken an overly narrow approach to defining pandemics, with the majority of attention focusing on the need for better surveillance and monitoring of emerging zoonotic infections.^4^

While zoonoses may indeed play a role in the next pandemic, comprehensive pandemic preparedness must involve planning for all potential pandemic sources: zoonoses, antimicrobial resistance (AMR), accidental release, and deliberate release.^5^ While deliberate release is already addressed through the *Biological Weapons Convention*,^6^ an inclusive global pandemic treaty must include provisions to tackle the other three main pandemic sources. Unfortunately, current discussions of a proposed treaty have focused on zoonoses and, to a lesser extent, accidental release, while completely ignoring AMR — a global health threat that is expected to result in USD $120 billion in excess hospital costs and potentially tens of millions of deaths by 2050.^7^ AMR is a natural process wherein pathogens evolve to become resistant to the antimicrobial medicines that are intended to treat them. Unlike acute disease threats, AMR is an ongoing evolutionary process that requires continuous management. This trait means AMR may appear to be a slower moving challenge than many zoonotic infections, but resistant pathogens already kill 700,000 people annually — and are getting worse each year.^8^ Managing the crisis of AMR will require global cooperation that can best be achieved through the robust coordination and accountability mechanisms offered under global health law.^9^ The potential negotiation of a pandemic treaty is the right time and appropriate context to ensure that effective global governance arrangements are in place to meaningfully address AMR in any emerging global health security instrument.

While the global governance of AMR requires unique legal considerations that may not all apply to zoonoses and accidental release, there are many important actions that overlap across pandemic sources (Figure 1).^10^ This overlap highlights the opportunity to develop regulatory strategies that proactively address all pandemic sources simultaneously rather than responding reactively to each type of threat in isolation. To address the threat of AMR alongside other pandemic threats, three major areas for action will be needed: 1) global intersectoral cooperation; 2) equitable resource allocation; and 3) strengthened accountability mechanisms.

To address the threat of AMR alongside other pandemic threats, three major areas for action will be needed: 1) global intersectoral cooperation; 2) equitable resource allocation; and 3) strengthened accountability mechanisms.

**Figure 1:**
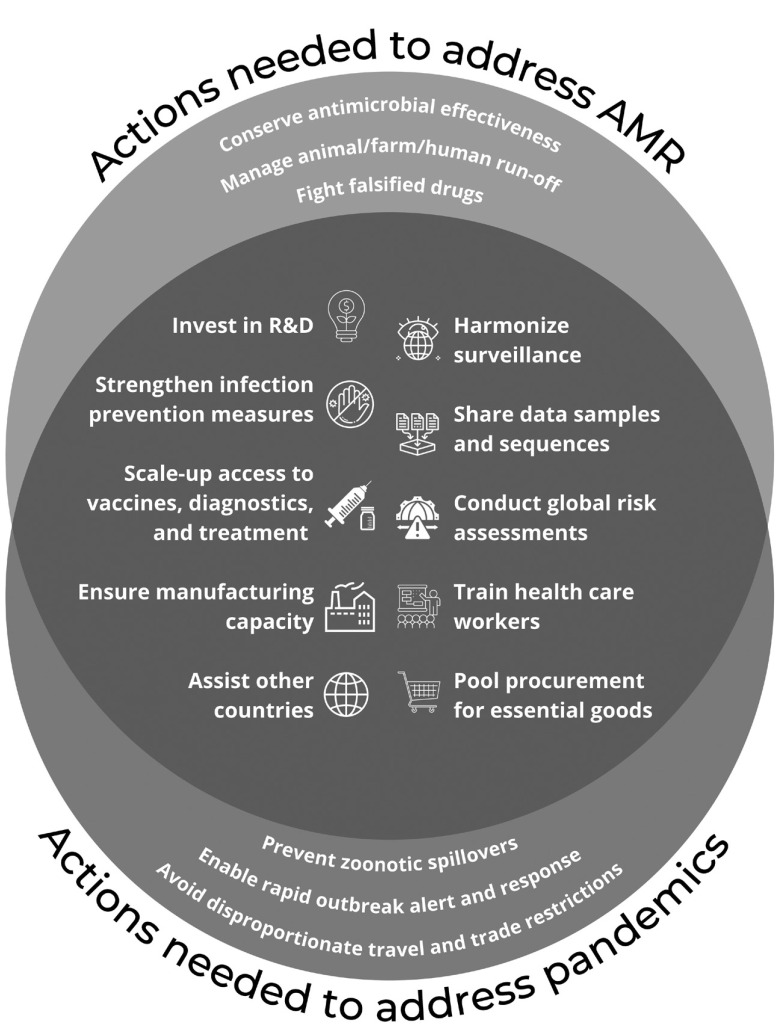
Strategies to mitigate the impact of AMR and other sources of future pandemics

## Global Intersectoral Cooperation

Preparing for AMR and zoonotic pandemics will require significant coordination across human, animal, and environmental health sectors, as well as within and among countries. Given the ease with which pathogens can cross national borders, countries are incentivized to ensure that each individual country can address outbreaks before they spread.^11^ While interconnections between countries and sectors may facilitate the spread of disease, they can also facilitate the sharing of knowledge and innovation; a new strategy, technology, or antimicrobial can benefit all parties, provided these innovations are shared globally. A well-designed treaty that addresses AMR and other pandemics should incentivize the sharing of these innovations through global health governance to ensure that shared vulnerabilities are minimized while simultaneously strengthening preparedness across countries. Despite the emphasis currently being placed on averting future zoonoses, our lack of preparedness for the COVID-19 pandemic is a reminder that we cannot be sure what the source of the next pandemic will be or which sectors it will impact. A proactive plan that is enshrined in international law and comprehensively accounts for *all* potential pandemic sources will help to bolster global efforts to respond quickly and effectively.

## Equitable Resource Allocation

Many of the countries most impacted by global health threats are also among the poorest, making it particularly challenging, if not unrealistic, for them to bear the full financial burden of a pandemic alone. Furthermore, in our globalized world, action on the part of low-income countries inherently benefits high-income countries, which may raise concerns about equity when the burdens and benefits accrued from action against health threats are unfairly distributed.^12^ These realities disincentivize cooperation and may generate nationalist actions, undermining the global solidarity necessary in a global response. The core capacities for mounting an effective response to AMR and other global health threats are extremely similar across pandemic sources — e.g., sanitation and hygiene for infection prevention; procurement of personal protective equipment; access to vaccines, diagnostics, and treatment^13^ — but current international legal mechanisms do not enable the global pooling of resources that would be required for all countries to meet their needs. Thus, in addition to offering an efficient means of simultaneously mitigating the harms associated with AMR and other global health threats, a comprehensive pandemic treaty that supports resource pooling can strengthen overall global pandemic preparedness while also promoting global health equity.

## Strengthened Accountability Mechanisms

The current system of global governance presents many challenges and incentive structures that hinder cooperation in global health. The COVID-19 pandemic has revealed that existing international legal frameworks do not incentivize cooperation with clear regulations, lack accountability mechanisms for those who do not comply, and provide inadequate support for those who are unable to fully implement them.^14^ Like the COVID-19 pandemic response, previous efforts to manage the global antimicrobial commons have also suffered from a lack of effective surveillance and enforcement that would enable the early identification of new threats and opportunities.^15^ Harmonized monitoring and accountability mechanisms that are simple, robust, transparent, and responsive are needed for all global health threats. A comprehensive and well-designed pandemic treaty should provide these mechanisms so that they can be applied to any of the main pandemic sources, regardless of the perceived speed at which they move.

## Conclusion

Many of the challenges hindering the global governance of AMR are the same challenges that must be overcome to address future zoonotic pandemics. COVID-19 has offered an unprecedented opportunity to evaluate the ways in which we approach global health threats under global health law, but early discussions of a global pandemic treaty remain narrowly focused on zoonotic diseases, with insufficient attention to other pandemic sources. In order for this treaty to be robust and comprehensive, AMR must be addressed in it as well. If AMR has to remain outside the scope of the treaty’s core content for political or logistical reasons, the treaty should have a mechanism for negotiating legally binding protocols on different issues that can be applied to a broader range of global health threats that are not addressed in the treaty’s main text. If that happens, an AMR-specific protocol should be among the first protocols to be developed in order to build quickly the necessary global governance arrangements needed to redress this growing crisis. A policy window is currently open to meaningfully address both AMR and other global pandemics, and the world should seize the opportunity to enact real change.

## Note

Steven J. Hoffman is funded by the Canadian Institutes of Health Research and the Ontario Ministry of Research, Innovation & Science.
